# Generalized estimating equations for modeling cluster randomized trial data on smoking cessation among tuberculosis patients

**DOI:** 10.1371/journal.pone.0333992

**Published:** 2025-10-10

**Authors:** Vasantha Mahalingam, Ratnakar Singh, Ramesh Kumar Santhanakrishnan, Adhin Bhaskar, Ponnuraja Chinnaiyan

**Affiliations:** 1 Department of Statistics, ICMR-National Institute for Research in Tuberculosis, Chennai, Tamil Nadu, India; 2 Department of Clinical Research, ICMR-National Institute for Research in Tuberculosis, Chennai, Tamil Nadu, India; Cytel Health Canada Inc., CANADA

## Abstract

There is a paucity of studies applying Generalized Estimating Equations (GEE) for longitudinal analysis of smoking cessation outcomes within the framework of a cluster randomized trial, especially among tuberculosis (TB) patients. In this study, a GEE model which accounts for repeated measures and cluster-level effects was implemented to identify factors associated with smoking cessation among TB patients. The data included 375 TB patients who were smokers and given TB treatment during 2013–2016 in Kanchipuram and Villupuram districts under a cluster randomized trial. GEE modeling provided robust, population-averaged estimates while accounting for intra-cluster correlation, confirming the sustained impact of these interventions. The model demonstrated that smoking cessation interventions, when integrated with TB treatment, had an impact on cessation outcomes in these populations.

## 1 Introduction

Smoking is highly prevalent among tuberculosis (TB) patients and is associated with poor treatment adherence and outcomes [1–4]. The global population of tobacco smokers aged ≥15 years was estimated to be around 0.96 billion by 2025, based on country wise prevalence and trends in population growth [5]. Of the 1.91 million (75%) TB patients in India who underwent tobacco use screening in 2023, 0.213 million (11%) were found to be tobacco users [6]. In India, approximately 32% of TB-related deaths attributed to bidi smoking [7]. The general strategies recommended for smoking cessation are doctor’s advice, nicotine replacement therapies (NRTs) and use of drug therapy. Pharmacological smoking cessation interventions in TB patients are critical due to the harmful impact of smoking on TB treatment outcomes and general health. A study from South Africa revealed that smoking habit was one of the significant factor associated with TB using binary logistic regression model [8]. Numerous studies have demonstrated the effectiveness of pharmacological interventions in aiding TB patients to quit smoking [9]. A randomized controlled trial from India showed that patients who received NRT combined with behavioral counseling had higher quit rates than those who received counseling only [10]. In the realm of smoking cessation, both NRT and Bupropion Sustained Release (SR) are recognized as effective pharmacological interventions [11]. Clinical studies indicate that Bupropion SR can double the likelihood of quitting smoking compared to placebo, with reported quit rates ranging from approximately 19% to 24% at six months post-treatment [12,13]. In terms of cost-effectiveness, studies have shown that Bupropion SR may also present a more favorable economic profile compared to NRT, particularly when considering the long-term health benefits associated with successful smoking cessation [14,15]. Prior studies in India have shown encouraging results for Bupropion SR in the general population [16,17]. Furthermore, if there is an addition of behavioral support to the treatment, chances to sustain quitting also increase [18–20]. Evidence shows that the most receptive period for cessation advice for TB patients is during treatment because the TB patients are more aware of their health status and the effects of smoking [21]. Moreover, the implementation of smoking cessation programs within TB treatment frameworks can lead to improved health outcomes. Evidence indicates that smoking cessation improves TB treatment outcomes while also lowering the risk of relapse and re-infection [22].

Generalized Estimating Equations (GEE) is a statistical method used to analyze correlated data, such as repeated measurements on the same individuals or data clustered within groups. GEE extends generalized linear models to account for within-subject or within-cluster correlation, providing robust, population-averaged estimates of associations between predictors and outcomes. GEE is used to identify covariates in a longitudinal study in which same parameters were measured at different time points. It has been particularly useful in cases where the distribution of the outcome variable is not known or when the focus is on the marginal effects of covariates on the outcome [23]. GEE was used to assess trends in smoking behavior, including present and passive cigarette smoking as well as hookah use, over time in a lifestyle intervention for adolescents [24]. Another study employed GEE to probe factors affecting sources of knowledge on smoking and awareness of smoking associated diseases among male urban secondary school students [25]. One of the key advantages of GEE is that it gives consistent estimates of the regression coefficients even when the correlation matrix is not correctly specified [20,26,27]. Our center ICMR-National Institute for Research in Tuberculosis (NIRT) conducted a cluster-randomized trial (CRT) on smoking cessation among TB patients aged ≥18 years who were smokers, comparing Bupropion SR and Enhanced Counseling (EC) with standard counseling for smoking cessation [28]. The trial results indicated that both EC and Bupropion SR were effective strategies for smoking cessation based on unadjusted statistical analysis. In India, there is a paucity of studies applying GEE for longitudinal data analysis of smoking cessation outcomes within the framework of a CRT, especially among TB patients. We applied a GEE model to evaluate smoking cessation outcomes, as it accounts for both repeated measures within individuals and clustering of patients within TB treatment centers inherent in the CRT. GEE provides robust, population-averaged estimates of intervention effects, making it appropriate for assessing overall effectiveness of smoking cessation interventions among TB patients. This paper utilizes data on TB patients’ smoking consumption and integrates changes in smoking behavior across various socio-economic and demographic sub-groups from two districts in Tamil Nadu, India. This approach helps to identify the factors contributing to smoking cessation during and at the end of the TB treatment period.

## 2 Methods

### 2.1 Data source

The secondary data on 375 male TB patients (new and previously treated smear-positive cases) aged ≥18 years with current history of smoking and who were initiated on treatment under 36 National TB Elimination Programme (NTEP) centres (formerly the Revised National TB Programme (RNTCP)) in Kanchipuram and Villupuram districts of Tamil Nadu, India were considered for the study. This data was obtained from a CRT conducted at the ICMR-NIRT during 2013–2016. The details of design and other particulars can be found elsewhere [28]. The study was registered with the clinical trial registry of India (no CTRI/2013/07/003830). The centres were randomly assigned to one of the following interventions: Bupropion SR plus standard counseling, EC, or the Standard Counseling/Control arm. Socio-demographic and smoking-related details of the research participants were collected through a questionnaire, and the administered interventions were documented in the pre designed study forms. The variables age, education level, occupation type, marital status, age at started smoking, type of smoking, and level of nicotine dependence (measured by the Fagerstrom Index), reason for smoking were considered for the study. The status of smoking habit at the end of second month and at the end of TB treatment period were recorded. Smoking status was assessed based on self-reports and confirmed by carbon monoxide test (reading <10 parts per million (ppm) considered quit). In cases of discrepancies between self-reported status and test results, the final classification was ‘still smoking’ if either method indicated still smoking. Patients were classified as either ‘quit smoking’ or ‘still smoking’. Self-reported smoking status was a binary variable, categorized as ‘quit smoking’ or ‘still smoking’.

### 2.2 Statistical methods

The GEE method is often used to fit marginal models, where the association between the response and covariates is modeled separately from the correlation of repeated measurements within individuals [29].

#### 2.2.1 Fundamental concepts and structure of GEE.

The GEE method is derived from the Generalized Linear Model (GLM) to handle longitudinal data where responses are correlated and was developed by Liang and Zeger [30,31]. GEE is used on quasi-likelihood instead of maximum likelihood estimation (MLE) because it does not require a fully specified likelihood function for parameter estimation. Instead, it only assumes a working correlation structure for the repeated measurements while modeling the mean and variance relationships. MLE is commonly used for parameter estimation. However, for some distributions within the exponential family, a standard likelihood function may not be available. In such cases, the quasi-score method can be applied, where the model is based on mean and variance of the data. The quasi-score function forms the basis of the estimation procedure in the GEE framework. Therefore, parameter estimation in GEE relies on the quasi-likelihood approach rather than the traditional maximum likelihood method.

The model defines the marginal expectation of dependent variable as linear combination of independent variables. Specifically, the mean $E\left(Y_{ij}\right)$ has been estimated as a function of independent variables.

- The expected value of dependent variable given the covariates, denoted as $E(Y_{ij}|X_{ij}=\;\mu_{ij}$ is linked to the covariate vector $x_{ij}$ through the equation, $g(\mu_{ij}=\;x_{ij}\beta$. g(. ) which uses a link function appropriate to the distribution of the dependent variable.
- Variance of $Y_{ij}$ is a function of its mean, expressed as $Var(Y_{ij}=\;\varphi v(\mu_{ij})$, where v(. ) is determined by distribution of the dependent variable (response) and φ be a parameter scale that must be estimated.
- The relation between two responses, $y_{ij}$ and $y_{ik}$ is expressed in terms of their marginal estimated average and a correlation parameter expressed as $\;corr(y_{ij},\;y_{ik}=\;\psi (\mu_{ij};\;\mu_{ik};\;\alpha$), where ψ (.) is a specified correlation function and α denotes the correlation parameter.

Assumptions in GEE are:

(a) The dependent variables are correlated within clusters
(b) Homogeneity of variance (constant variance) is not required
(c) The independent variables do not exhibit multicollinearity
(d) The correlation structure among repeated measurements must be specified or estimated
(e) Parameter estimation is based on quasi-likelihood rather than full likelihood methods

The components of GEE model, which extend the GLM are given as:

- The dependent variable $\left(y_{ij}\right)$ follows distribution from the exponential family, where i=1,2,…, N denotes $i^{th}$ subject and $\;j=\text{1},\text{2},\ldots ,\;n_{t},$ j denotes the repeated measurement for each subject.
- The linear predictor is defined as $\eta_{ij}=x_{ij}^{T}\beta \;$ where $x_{ij}$ is the covariate vector and β is the vector of regression coefficients.
- A known link function g(· relates the mean of the response variable, $\mu_{ij}=E(Y_{ij}|X_{ij}$ to the linear predictor such that $g(\mu_{ij}=\;\eta_{ij}$

#### 2.2.2 GEE estimation.

If yij is the dependent variable for $i^{th}$ subject where observations are made over T time points for each of n subjects, i=1,2, .... n. $x_{it}$ is an associated covariate vector of dimension p for all yij, The response vector can be denoted as $y_{i}$ and covariate matrix is represented as $X_{i}$ for each subject. It is assumed that the data pairs $\left(y_{i}\;,\;X_{i}\right)$ are independent and identically distributed across subjects.


$$E\left(y_{ij}\right|X_{i})\;=\;E\left(y_{ij}\right|x_{it})\;=\;x_{it}^{T}\beta$$ (1)

The GEE U(β) equation can be written as [32]:


$$\text{U}(\beta )={\left[{\left\{\left(\sum {\mathrm{\backslash nolimits}}_{i=\text{1}}^{N}x_{mi}^{T}D\left(\frac{\partial \mu }{\partial \eta }\right)\left[V{\left(\mu_{i}\right)}^{-\text{1}}\left(\frac{y_{i}-\mu_{i}}{a(\varphi )}\right)\right]\right)\right\}}_{m=\text{1},\ldots ,p}\right]}_{\left(\text{p}\times \text{1}\right)}=[\text{0}{]}_{\left(\text{p}\times \text{1}\right)}\;$$ (2)

D(.) denote the matrix derived from the derivative of the mean with respect to the model parameters. The term a(ϕ) represents a function of the dispersion parameter ϕ typically used to account for over dispersion in the data. $V\left(\mu_{i}\right)$ is a diagonal matrix of independent correlation structure from the form:


$$V\left(\mu_{i}\right)={\left[D{\left(V\left(\mu_{ij}\right)\right)}^{\frac{\text{1}}{\text{2}}}I_{{(n}_{t}xn_{t})}D{\left(V(\mu_{ij})\right)}^{\frac{\text{1}}{\text{2}}}\right]}_{n_{t}xn_{t}}$$ (3)

However, for different structure of correlation, the variance-covariance matrix $V(\mu_{i})$ for the $i^{th}$ subject can generally be expressed as:


$$V\left(\mu_{i}\right)={\left[D{\left(V\left(\mu_{ij}\right)\right)}^{\frac{\text{1}}{\text{2}}}R(\alpha {)}_{\left(n_{t}xn_{t}\right)}D{\left(V(\mu_{ij})\right)}^{\frac{\text{1}}{\text{2}}}\right]}_{n_{t}xn_{t}}$$ (4)

R(α)denotes the correlation matrix which is calculated using the parameter vector α.

The equation for estimating the regression coefficients in a GEE model is [33]:


$$U(\beta )=\sum {\mathrm{\backslash nolimits}}_{i=\text{1}}^{N}X_{i}^{T}V_{i}^{-\text{1}}[Y_{i}-g\left(X_{i}\beta \right)]=\text{0}$$ (5)

where $Y_{i}$ is $i^{th}$ observation vector (the response variable for the i-th subject or cluster), $X_{i}$ is the $i^{th}$ design matrix (the matrix of independent variables for the $i^{th}\;$ observation), β is the vector of unknown parameters (the regression coefficients to be estimated), $g(X_{i}\;\beta )$ is the regression function, where g(·) is the link function (e.g., identity, logit, etc.), $V_{i}$ is the covariance matrix of the $i^{th}\;$ observation, which accounts for the within-subject correlation. The structure of $V_{i}$ depends on the assumed correlation structure, $V_{i}$ reflects the assumed correlation between the repeated measurements within the same subject: Exchangeable (constant correlation between measurements) or autoregressive (correlation decays with time or distance) or Unstructured (no assumption about the correlation). In GEE, robust standard errors are used to provide valid inference even if the correlation structure is misspecified [31]. The estimation is done by solving the GEE equation, which is usually achieved through iterative methods, such as the iterative weighted least squares algorithm [34].

#### 2.2.3 GEE for binary data.

When the outcome variable $Y_{it}$ is binary, the GEE model is specified using a logistic link function with a binomial distribution. For a binary outcome variable $\;Y_{it}$, the GEE model is expressed as [35]:


$$logit(\pi_{it})=log\left(\frac{\pi_{it}}{\text{1}-\pi_{it}}\right)=\beta_{\text{0}}+\beta_{\text{1}}X_{\text{1}it}+\beta_{\text{2}}X_{\text{2}it}+\cdots +\beta_{p}X_{pit}$$ (6)

where, $\pi_{it}=P(Y_{it}=\text{1}|X_{it})$ is the probability of quitting smoking at time t for individual i, $X_{\text{1}it},X_{\text{2}it},...,X_{pit}\;$ are the predictor variables, $\beta_{\text{0}}$ is the intercept, and $\beta_{\text{1}},\beta_{\text{2}},...,\beta_{p}$ are the regression coefficients, the logit link function $log\left(\frac{\pi }{\text{1}-\pi }\right)$ ensures that the model predicts probabilities between 0 and 1.

#### 2.2.4 Estimation of regression coefficients.

For estimating the regression coefficients in a GEE model, $g(X_{i}\beta )$ is the expected value of $\;{\;Y}_{i}$, given by the logistic function given below (7):


$$g\left(X_{i}\beta \right)=\frac{exp\left(X_{i}\beta \right)}{\text{1}+exp\left(X_{i}\beta \right)}$$ (7)

where, $V_{i}$ is the working variance-covariance matrix, which accounts for within-subject correlation. It is defined as:


$$V_{i}=A_{i}^{\text{1}/\text{2}}\;R(\alpha )A_{i}^{\text{1}/\text{2}}\;$$ (8)

where, $A_{i}$ is a diagonal matrix with elements $\;\left(\frac{\pi_{it}}{\text{1}-\pi_{it}}\right)$, representing the variance of the binary outcome and R(α) is the working correlation matrix, specifying the assumed correlation structure within subjects.

#### 2.2.5 Covariance matrix in the model.

In GEE framework, two types of variance-covariance estimator matrices are commonly used: the naïve estimator and the robust (or sandwich) estimator. These estimators serve two primary purposes: (i) to assess the statistical significance of covariates in the model, and (ii) to evaluate the appropriateness of the predefined correlation pattern. The mathematical forms of the Equations (9) and (11) show the naïve and robust variance estimators, respectively:


$$V\left(\hat{\beta }\right)={\left[\sum {\mathrm{\backslash nolimits}}_{i=\text{1}}^{N}D_{i}^{T}\left({\hat{V}}_{i}^{-\text{1}}\right)D_{i}\right]}^{-\text{1}}$$ (9)

for data with normal distribution, $D_{i}=X_{i}$, so


$$V\left(\hat{\alpha }\right)={\left[\sum {\mathrm{\backslash nolimits}}_{i=\text{1}}^{N}X_{i}^{T}\left({\hat{V}}_{i}^{-\text{1}}\right)X_{i}\right]}^{-\text{1}}$$ (10)

Naïve estimator will be useful for small sample size (N < 20).


$$V\left(\hat{\beta }\right)=\sum {\mathrm{\backslash nolimits}}_{i=\text{1}}^{N}M_{\text{0}}^{-\text{1}}M_{\text{1}}M_{\text{0}}^{-\text{1}}$$ (11)

with


$$M_{\text{0}}=\left[\sum {\mathrm{\backslash nolimits}}_{i=\text{1}}^{N}D_{i}^{T}\left({\hat{V}}_{i}^{-\text{1}}\right)D_{i}\right]$$ (12)

$$M_{\text{1}}=\left[\sum {\mathrm{\backslash nolimits}}_{i=\text{1}}^{N}D_{i}^{T}\left({\hat{V}}_{i}^{-\text{1}}\right)\left(y_{i}-{\hat{\mu }}_{i}\right){\left(y_{i}-{\hat{\mu }}_{i}\right)}^{T}D_{i}\right]$$ (13)

For large sample size, robust variance estimator is appropriate.

#### 2.2.6 Correlation structure of the GEE model.

The correlation pattern is defined as $\;R_{i}(\alpha )$, here α represents the average dependence between repeated measurements within the same subjects. The estimate of this parameter, denoted$\;\hat{\alpha }$, quantifies the correlation structure for each pair of repeated observations. Before evaluating the correlation pattern, the residual error is calculated using equation (14):


$$e_{ij}=\frac{{(y}_{ij}-\mu_{ij})}{\sqrt{\frac{v(\mu_{ij})}{\phi }}}$$ (14)

The error, ${\hat{e}}_{ij}$ is applied for getting values of $\hat{\alpha }$ and ϕ will be useful to predict correlation. Suppose the data exhibit over dispersion, then the dispersion parameter ϕ is estimated using equation (15):


$$\phi =\frac{\text{1}}{K-p}\sum {\mathrm{\backslash nolimits}}_{i=\text{1}}^{N}\sum {\mathrm{\backslash nolimits}}_{j=\text{1}}^{n_{t}}e_{ij}^{\text{2}}$$ (15)

Here, $K=\sum_{i=\text{1}}^{N}n_{i}$ and p is the number of covariates.

#### 2.2.7 Parameter estimation of the model.

Parameters calculation in the model involves solving equation (5), which requires prior knowledge of the assumed distribution of the longitudinal data. The first step is to identify the shape of the data distribution, which is then expressed in the form of an exponential family distribution. From this, the mean and variance functions are derived, which are central to constructing the GEE.

For example, if the longitudinal response data are assumed to follow a normal distribution, the corresponding exponential family form is expressed as:


$$f\left(y|\mu ,\sigma^{\text{2}}\right)=\frac{\text{1}}{{\left(\text{2}\pi \sigma^{\text{2}}\right)}^{\frac{\text{1}}{\text{2}}}}\text{exp}\left[-\frac{\text{1}}{\text{2}\sigma^{\text{2}}}(y-\mu {)}^{\text{2}}\right]$$ (16)

$$=\text{exp}\left\{ln\left({\left(\text{2}\pi \sigma^{\text{2}}\right)}^{-\frac{\text{1}}{\text{2}}}\right)-\left[\frac{\text{1}}{\text{2}\sigma^{\text{2}}\left(y-\text{2}y\mu +\mu^{\text{2}}\right)}\right]\right\}$$ (17)

$$=\text{exp}\left\{\frac{y\mu -\frac{\text{1}}{\text{2}}\mu^{\text{2}}}{\sigma^{\text{2}}}+\left({ln\left(\text{2}\pi \sigma^{\text{2}}\right)}^{-\frac{\text{1}}{\text{2}}}\right)-\frac{y^{\text{2}}}{\text{2}\sigma^{\text{2}}}\right\}$$ (18)

This has been observed that $\theta =\mu ,\;b(\theta \;)=\frac{\text{1}}{\text{2}}\mu^{\text{2}}$ and $\;(\phi )=\sigma^{\text{2}}$. When the dependent variable follows normal distribution with mean = 0 and variance = 1, then the variance is:


$$V\left(\mu_{it}\right)=(\begin{array}{ccccc} \text{1} & \text{0} & \cdots & \text{0} & \text{0}\\ \text{0} & \text{1} & \text{0} & \text{0} & \text{0}\\ \vdots & \text{0} & \ddots & \text{0} & \vdots \\ \vdots & \vdots & \vdots & \text{1} & \text{0}\\ \text{0} & \text{0} & \cdots & \cdots & \text{1} \end{array})$$ (19)

The variance of the model for the normal distribution can be derived as given below:


$$V\left(\hat{\alpha }\right)=\phi A_{i}^{\frac{\text{1}}{\text{2}}}R_{i}\left(\hat{\alpha }\right)A_{i}^{\frac{\text{1}}{\text{2}}}$$ (20)

$$=(\begin{array}{ccccc} \text{1} & \text{0} & \cdots & \text{0} & \text{0}\\ \text{0} & \text{1} & \text{0} & \text{0} & \text{0}\\ \vdots & \text{0} & \ddots & \text{0} & \vdots \\ \vdots & \vdots & \vdots & \text{1} & \text{0}\\ \text{0} & \text{0} & \cdots & \cdots & \text{1} \end{array})R_{i}(\alpha )(\begin{array}{ccccc} \text{1} & \text{0} & \cdots & \text{0} & \text{0}\\ \text{0} & \text{1} & \text{0} & \text{0} & \text{0}\\ \vdots & \text{0} & \ddots & \text{0} & \vdots \\ \vdots & \vdots & \vdots & \text{1} & \text{0}\\ \text{0} & \text{0} & \cdots & \cdots & \text{1} \end{array})$$

$$=\phi R_{i}(\hat{\alpha })$$

Thus, the model equation for longitudinal data that follows a normal distribution is given by (21):


$$\sum {\mathrm{\backslash nolimits}}_{i=\text{1}}^{N}X_{i}^{T}{\left[\phi R_{i}\left(\hat{\alpha }\right)\right]}^{-\text{1}}(y_{i}-X_{i}\beta )=\text{0}$$ (21)

This is split down into:


$$\sum {\mathrm{\backslash nolimits}}_{i=\text{1}}^{N}X_{i}^{T}{\left[\phi R_{i}\left(\hat{\alpha }\right)\right]}^{-\text{1}}y_{i}-\sum {\mathrm{\backslash nolimits}}_{i=\text{1}}^{N}X_{i}^{T}{\left[\phi R_{i}\left(\hat{\alpha }\right)\right]}^{-\text{1}}X_{i}\beta =\text{0}$$ (22)

$$\sum {\mathrm{\backslash nolimits}}_{i=\text{1}}^{N}X_{i}^{T}{\left[\phi R_{i}\left(\hat{\alpha }\right)\right]}^{-\text{1}}y_{i}=\sum {\mathrm{\backslash nolimits}}_{i=\text{1}}^{N}X_{i}^{T}{\left[\phi R_{i}\left(\hat{\alpha }\right)\right]}^{-\text{1}}X_{i}\beta$$ (23)

$${\hat{\beta }=\left[\sum {\mathrm{\backslash nolimits}}_{i=\text{1}}^{N}X_{i}^{T}{\left[\phi R_{i}\left(\hat{\alpha }\right)\right]}^{-\text{1}}X_{i}\right]}^{-\text{1}}\left[\sum {\mathrm{\backslash nolimits}}_{i=\text{1}}^{N}X_{i}^{T}{\left[\phi R_{i}\left(\hat{\alpha }\right)\right]}^{-\text{1}}y_{i}\right]$$ (24)

In general, $\hat{\beta }$ is calculated using the below equation (25):


$${\hat{\beta }=\left[\sum {\mathrm{\backslash nolimits}}_{i=\text{1}}^{N}X_{i}^{T}{\left[\phi R_{i}\left(\hat{\alpha }\right)\right]}^{-\text{1}}X_{i}\right]}^{-\text{1}}\left[\sum {\mathrm{\backslash nolimits}}_{i=\text{1}}^{N}X_{i}^{T}{\left[\phi R_{i}\left(\hat{\alpha }\right)\right]}^{-\text{1}}y_{i}\right]$$ (25)

The estimation of the $\hat{\beta }$ parameter in the GEE model is performed numerically to obtain a convergent solution.

### 2.3 Ethics

Since this modeling study involved secondary data analysis, acquiring ethical approval was not applicable. The original study was ethically approved and written informed consent were obtained from the study participant [28]. This study data allowed for a comprehensive assessment of the covariates affecting the effectiveness of each intervention in promoting smoking cessation. The analysis was conducted using the IBM SPSS Statistics for Windows Version 25.0 (IBM Corp., Armonk, NY, USA).

## 3 Results

Of 375 TB patients, all males, 198 (52.8%) were aged >45years, 282 (75.2%) were literate, 352 (93.9%) were employed. Additionally, 320 (85.3%) were married, 226 (60.3%) began smoking at or before the age of 20 years. Among 375 patients, 120 (32%) were received the intervention Bupropion SR + Standard counselling, 114 (30.4%) were received the intervention EC and 141 (37.6%) recruited the control arm standard counselling. Among the 375 patients, 215 (57.3%) and 247(65.9%) stopped smoking after 2^nd^ and 6^th^ months respectively while 160 (42.7%) and 128 (34.1%) were still smoking after 2^nd^ and 6^th^ months respectively. The reasons for smoking habits among participants were categorized as follows: The majority, 243 TB patients (64.8%), reported that they were smoking in the company of friends, while 72 patients (19.2%) were smoking out of curiosity or the desire to experience tobacco use. Additionally, 105 (28%) smoked to cope with for emotional distress. Table 1 shows the basic characteristics of TB patients based on their smoking cessation status at the end of treatment.

**Table 1:** Comparison of basic characteristics of TB patients based on smoking cessation status.

Variables	Smoking Status n (%)		Total (%) N = 375
	Quit Smoking n = 247	Still Smoking n = 128	
**Age in years**			
> 45	114 (46.2)	63 (49.2)	177 (47.2)
≤ 45	133 (53.8)	65 (50.8)	198 (52.8)
**Education**			
Literate	188 (76.1)	94 (73.4)	282 (75.2)
Illiterate	59 (23.9)	34 (26.6)	93 (24.8)
**Employment**			
Unemployed	16 (6.5)	7 (5.5)	23 (6.1)
Employed	231(93.5)	121(94.5)	352 (93.9)
**Marital Status**			
Unmarried	42 (17.0)	13 (10.2)	55 (14.7)
Married	205 (83.0)	115 (89.8)	320 (85.3)
**Age of start smoking in years**			
> 20	101(40.9)	48 (37.5)	149 (39.7)
≤ 20	146 (59.1)	80 (62.5)	226 (60.3)
**Type of smoking**			
Others	2 (0.8)	1 (0.8)	3 (0.8)
Beedi & Cigarette	45 (18.2)	32 (25.0)	77 (20.5)
Beedi	114 (46.2)	58 (45.3)	172 (45.9)
Cigarette	86 (34.8)	37 (28.9)	123 (32.8)
**Fagerstom index**			
Low dependent	192 (78.4)	87 (68.0)	279 (74.8)
High dependent	53 (21.6)	41 (32.0)	94 (25.2)
**In the company of friend**			
No	94 (38.1)	38 (29.7)	132 (35.2)
Yes	153 (61.9)	90 (70.3)	243 (64.8)
**Wanted to experience**			
No	201(81.4)	102 (79.7)	303 (80.8)
Yes	46 (18.6)	26 (20.3)	72 (19.2)
**Emotional problem**			
No	174 (70.4)	96 (75.0)	270 (72.0)
Yes	73 (29.6)	32 (25.0)	105 (28.0)
**Patients randomized to treatment**			
Bupropion SR + Standard counselling	81(32.8)	39 (30.5)	120 (32.0)
Enhanced counselling	94 (38.1)	20 (15.6)	114 (30.4)
Standard counselling	72 (29.1)	69 (53.9)	141(37.6)

GEE was fitted to identify the factors associated with quit smoking over time points and the estimated effects in terms of odds ratio (OR) are presented with 95% confidence interval (CI) in Table 2. The quit smoking was significantly higher among the patients who received the Bupropion SR plus standard counselling (OR: 1.86, 95%C.I (1.17, 2.94), p < 0.01) and even greater in patients who received EC (OR: 3.26, 95% C.I (1.99, 5.34), p < 0.001) compared to those patients in the control group receiving standard counselling alone. Additionally, smoking cessation rates increased significantly by the end of the sixth month of treatment (OR: 1.48, 95% CI: 1.20–1.82, p < 0.001) compared to baseline.

**Table 2:** Parameter estimates of GEE model for factor influencing smoking cessation among TB patients.

Variable	Estimate	Standard Error	Exp(B)	95% Confidence Interval for Exp(B)		p-value
				Lower	Upper	
**Age in years**						
> 45	0.143	0.203	1.154	.776	1.716	0.480
<=45	Reference					
**Education**						
Literate	0.029	0.231	1.030	1.030	.655	0.899
Illiterate	Reference					
**Employment**						
Unemployed	.378	0.425	1.459	.634	3.360	0.374
Employed	Reference					
**Marital Status**						
Unmarried	0.284	0.278	1.329	.771	2.290	0.306
Married	Reference					
**Age at start smoking in years**						
> 20	.030	0.200	1.030	.697	1.524	0.881
<=20	Reference					
**Type of smoking**						
Others than beedi & Ciagartte	0.314	1.072	1.368	.167	11.183	0.770
Beedi & Cigratte	−0.296	0.285	.744	.425	1.300	0.299
Beedi	−0.204	0.238	.816	.511	1.301	0.392
Cigarette	Reference					
**Fagerstom index**						
Low dependent	0.274	0.229	1.315	.840	2.059	0.231
High dependent	Reference					.
**In the company of friend**						
No	0.407	0.251	1.503	.920	2.455	0.104
Yes	Reference					
**Wanted to experience**						
No	0.266	0.288	1.305	.742	2.295	0.356
Yes	Reference					
**Emotional problem**						
No	0.342	0.235	1.408	.888	2.233	0.146
Yes	Reference					
**Patients randomized to treatment**						
Bupropion SR + Standard counselling (Arm T1)	0.620	0.235	1.859	1.174	2.943	p < 0.01
Enhanced Counselling (Arm T2)	1.182	0.251	3.260	1.991	5.336	p < 0.001
Standard Counselling (Arm C)	Reference					
**Time**						
At end of 6^th^ month of treatment	0.391	0.106	1.478	1.200	1.820	p < 0.001
At end of 2^nd^ month of treatment	Reference					

## 4 Discussion

In this study, we developed a GEE model to assess the effectiveness of EC and Bupropion SR on smoking cessation among TB patients in a CRT. A major strength of this study is the use of secondary data derived from a CRT design, which minimizes contamination between treatment groups and enhances the generalizability of findings. GEE was chosen as it accounts for the correlation among repeated observations within clusters and provides population-averaged estimates of treatment effects. Our results support the integration of EC approaches into TB treatment programs to maximize smoking cessation rates. The study findings indicate that smoking cessation rates were highest among patients who received EC, followed by those who received Bupropion SR plus standard counseling, compared to the control group with standard counseling alone. The likelihood of quitting smoking was significantly greater in the EC group, demonstrating its superior effectiveness. Additionally, cessation rates significantly improved over time, with a notable increase at the end of the 6th month compared to baseline.

Our study achieved a 57.3% cessation rate at the end of month 2 and a 65.9% cessation rate at the end of month 6 which is similar to reports of cessation intervention for tobacco use among TB patients in Sudan, Indonesia and Bangladesh [36]. GEE models are used to find the covariates responsible for the outcome variable of interest is feasible according to the data which we are handling. We observed a significant and positive effect of treatment arms on smoking cessation. Our findings are consistent with our main study [28]. We identified that quitting smoking showed difference pattern on smoking cessation interventions. Specifically the 6-month follow-up was positively associated with smoking cessation, indicating that the intervention became more effective over time.

In a randomized clinical trial evaluating the efficacy of bupropion SR and individual counseling among adult daily smokers, the bupropion SR treatment significantly increased abstinence rates compared to placebo, while individual counseling alone did not show a significant effect in quit smoking. The combination of bupropion SR and counseling did not yield higher abstinence rates than bupropion SR alone [37]. A recent network meta-analysis of 103 randomized controlled trials evaluated combined effect of various behavioural and pharmacological interventions on smoking cessation. The study found that combining behavioural therapies with pharmacotherapy was effective in smoking treatment and was recommended in clinical practice [38]. These studies suggest that EC, when combined with pharmacotherapy, can be more effective than standard behavioural interventions alone in promoting smoking cessation.

This study provides insight into public health since it addresses the critical problem of smoking among TB patients, which aggravates the challenges of treatment and increases the period of recovery. The GEE model enables the study to provide a robust statistical approach in determining factors that influence smoking cessation, both individual and socio-environmental variables. Findings may inform targeted interventions and healthcare policies for better treatment outcomes for TB as well as reduce smoking-related complications. Thus, the current research contributes to the global health by providing information that helps improve control strategies of the disease and patient adherence to tobacco smoking cessation during treatment.

The limitation of the study is that while GEE adjusts for intra-cluster correlation, unmeasured confounders might still influence the outcomes [39]. Future research should explore longer follow-up durations to assess the sustainability of cessation outcomes and examine whether the combination of pharmacotherapy and counseling offers additional long-term benefits. The study findings may not be pertinent to female smokers, as there were none in the study.

## 5 Conclusion

This study highlights the effectiveness of EC and Bupropion SR in promoting smoking cessation among TB patients, demonstrating the superiority of EC in achieving higher quit rates. The GEE model was instrumental in analyzing treatment effects while accounting for the correlation of repeated measures within clusters. The model provided robust, population-averaged estimates, reinforcing the validity of our findings. Our results show that smoking cessation rates improved significantly over time, with the highest success observed at the 6-month follow-up, confirming the sustained impact of the interventions. The significant association between treatment arms and smoking cessation further supports the integration of EC into TB treatment programs to maximize patient outcomes. The use of the GEE model in this study was appropriate and effective in handling the clustered data structure, addressing intra-cluster correlation, and providing reliable inferences on treatment effectiveness. These findings contribute to public health strategies by guiding targeted interventions and policy decisions to improve smoking cessation among TB patients. Future research should continue utilizing GEE models for analyzing clustered and longitudinal data in similar intervention studies.

## Supporting information

S1 File This is a minimal dataset for this analysis. (XLSX)

## References

[1] Ariyothai N, Podhipak A, Akarasewi P, Tornee S, Smithtikarn S, Thongprathum P. Cigarette smoking and its relation to pulmonary tuberculosis in adults. Southeast Asian J Trop Med Public Health. 2004;35(1):219–27. 15272772

[2] Altet-Gômez MN, Alcaide J, Godoy P, Romero MA, Hernández del Rey I. Clinical and epidemiological aspects of smoking and tuberculosis: a study of 13,038 cases. The International Journal of Tuberculosis and Lung Disease: The Official Journal of the International Union Against Tuberculosis and Lung Disease. 2005;9(4):430–6. 15830749

[3] Sharma SK, Mohan A, Singh AD, Mishra H, Jhanjee S, Pandey RM, et al. Impact of nicotine replacement therapy as an adjunct to anti-tuberculosis treatment and behaviour change counselling in newly diagnosed pulmonary tuberculosis patients: an open-label, randomised controlled trial. Sci Rep. 2018;8(1):8828. doi: 10.1038/s41598-018-26990-5 29891957 PMC5995820

[4] Lancaster T, Stead LF. Individual behavioural counselling for smoking cessation. Cochrane Database Syst Rev. 2005;(2):CD001292. doi: 10.1002/14651858.CD001292.pub2 15846616

[5] WHO global report on trends in prevalence of tobacco use 2000-2025, fourth edition. Geneva: World Health Organization. 2021.

[6] Central TB Division. NTEP India TB report 2024. http://www.tbcindia.gov.in

[7] Leung CC, Li T, Lam TH, Yew WW, Law WS, Tam CM, et al. Smoking and tuberculosis among the elderly in Hong Kong. Am J Respir Crit Care Med. 2004;170(9):1027–33. doi: 10.1164/rccm.200404-512OC 15282201

[8] Maja TF, Maposa D. An Investigation of Risk Factors Associated with Tuberculosis Transmission in South Africa Using Logistic Regression Model. Infect Dis Rep. 2022;14(4):609–20. doi: 10.3390/idr14040066 36005268 PMC9408379

[9] Whitehouse E, Lai J, Golub JE, Farley JE. A systematic review of the effectiveness of smoking cessation interventions among patients with tuberculosis. Public Health Action. 2018;8(2):37–49. doi: 10.5588/pha.18.0006 29946519 PMC6012961

[10] Sahar N, Roy D, Priyadarshi S, Tangade P, Singh V. Nicotine replacement therapy: A blessing in disguise. Int J Appl Dent Sci. 2021;7(2):225–8. doi: 10.22271/oral.2021.v7.i2d.1212

[11] Tobacco Use and Dependence Guideline Panel. Treating Tobacco Use and Dependence: 2008 Update. Nih.gov. 2008. https://www.ncbi.nlm.nih.gov/books/NBK63952/

[12] McCarthy DE, Jorenby DE, Minami H, Yeh V. Treatment Options in Smoking Cessation: What Place for Bupropion Sustained-Release?. Clinical Medicine Therapeutics. 2009;1. doi: 10.4137/cmt.s2044

[13] Hays JT, Ebbert JO. Bupropion sustained release for treatment of tobacco dependence. Mayo Clin Proc. 2003;78(8):1020–4; quiz 1024. doi: 10.4065/78.8.1020 12911050

[14] Song F, Raftery J, Aveyard P, Hyde C, Barton P, Woolacott N. Cost-effectiveness of pharmacological interventions for smoking cessation: a literature review and a decision analytic analysis. Med Decis Making. 2002;22(5 Suppl):S26-37. doi: 10.1177/027298902237708 12369228

[15] Mahalingam V, Santhanakrishnan RK, Malaisamy M, Chelvanayagam K, Mathiyazhagan K, Bhaskar A, et al. Cost-effectiveness analysis for implementation of smoking cessation strategies at primary health care settings in Tamil Nadu. PLoS One. 2025;20(1):e0318013. doi: 10.1371/journal.pone.0318013 39879203 PMC11778769

[16] Singh P, Kumar R. Assessment of the effectiveness of sustained release Bupropion and intensive physician advice in smoking cessation. Lung India. 2010;27(1):11–8. doi: 10.4103/0970-2113.59262 20539765 PMC2878706

[17] Lee J, Choi J-Y. Texas hospitals with higher health information technology expenditures have higher revenue: A longitudinal data analysis using a generalized estimating equation model. BMC Health Serv Res. 2016;16:117. doi: 10.1186/s12913-016-1367-9 27048305 PMC4820871

[18] Lin Y, Wang L-X, Qiu L-X, Huang Q, Shu Q, Lin H-X, et al. A smoking cessation intervention among tuberculosis patients in rural China. Public Health Action. 2015;5(3):183–7. doi: 10.5588/pha.15.0025 26399289 PMC4576768

[19] Black N, Johnston M, Michie S, Hartmann-Boyce J, West R, Viechtbauer W, et al. Behaviour change techniques associated with smoking cessation in intervention and comparator groups of randomized controlled trials: a systematic review and meta-regression. Addiction. 2020;115(11):2008–20. doi: 10.1111/add.15056 32196796

[20] Stead LF, Carroll AJ, Lancaster T. Group behaviour therapy programmes for smoking cessation. Cochrane Database Syst Rev. 2017;3(3):CD001007. doi: 10.1002/14651858.CD001007.pub3 28361497 PMC6464070

[21] Lin Y, Dlodlo RA, Shu Q, Lin H, Huang Q, Meng X, et al. Outcomes of a smoking cessation intervention at follow-up after 5 years among tuberculosis patients in China. Tob Induc Dis. 2019;17:69. doi: 10.18332/tid/111539 31582957 PMC6770632

[22] Purushothama J, Badiger S, Olickal JJ, Kunkulol R, Kumar N, D’Souza N. Effectiveness of Nicotine Replacement Therapy on Smoking Cessation and Reduction Among Pulmonary Tuberculosis Patients - A Randomized Controlled Trial. Int J Prev Med. 2023;14:33. doi: 10.4103/ijpvm.ijpvm_3_22 37351046 PMC10284237

[23] Masihay-Akbar H, Amiri P, Rezaei M, Jalali-Farahani S, Cheraghi L, Momenan AA, et al. The Long-Term Effectiveness of a Multisetting Lifestyle Intervention on Tobacco-Related Habits in Adolescent Boys and Girls: Tehran Lipid and Glucose Study. J Sch Health. 2022;92(9):888–97. doi: 10.1111/josh.13193 35585677

[24] Xu X, Chen C, Abdullah AS, Sharma M, Liu H, Zhao Y. Knowledge about and sources of smoking-related knowledge, and influencing factors among male urban secondary school students in Chongqing, China. Springerplus. 2016;5(1):1879. doi: 10.1186/s40064-016-3589-z 27833838 PMC5081986

[25] Goetgeluk S, Vansteelandt S. Conditional generalized estimating equations for the analysis of clustered and longitudinal data. Biometrics. 2007;64(3):772–80. doi: 10.1111/j.1541-0420.2007.00944.x 18047524

[26] French B, Heagerty PJ. Marginal mark regression analysis of recurrent marked point process data. Biometrics. 2008;65(2):415–22. doi: 10.1111/j.1541-0420.2008.01076.x 18573132 PMC8014910

[27] Ito T, Sugasawa S. Grouped Generalized Estimating Equations for Longitudinal Data Analysis. Biometrics 2022. doi: 10.1111/biom.13718 35819419

[28] Ramesh Kumar S, Dolla C, Vasantha M, Menon PA, Venkatesan G, Venkatesan P. Strategies for Smoking Cessation (Pharmacologic Intervention versus Enhanced Motivation vs. Standard Motivation) in TB Patients under Treatment in the RNTCP, India - a Cluster - Randomized Trial. Indian Journal of Tuberculosis. 2020;67(1):8–14. doi: 10.1016/j.ijtb.2020.01.006 32192623

[29] Awalluddin AS, Wahyuni I, Nurmuslimah H. Analysis of Longitudinal Regression Model Using the Generalized Estimating Equation (GEE) for the Child Welfare Composite Index (CWCI) in West Java. In: Advances in Social Science, Education and Humanities Research, 2021. doi: 10.2991/assehr.k.210508.094

[30] Zeger SL, Liang K-Y. Longitudinal Data Analysis for Discrete and Continuous Outcomes. Biometrics. 1986;42(1):121. doi: 10.2307/2531248 3719049

[31] Wang M, Kong L, Li Z, Zhang L. Covariance estimators for generalized estimating equations (GEE) in longitudinal analysis with small samples. Stat Med. 2015;35(10):1706–21. doi: 10.1002/sim.6817 26585756 PMC4826860

[32] Hardin JW, Hilbe JM. Generalized Estimating Equations. Chapman and Hall/CRC. 2002. doi: 10.1201/9781420035285

[33] Zeger SL, Liang K-Y, Albert PS. Models for Longitudinal Data: A Generalized Estimating Equation Approach. Biometrics. 1988;44(4):1049. doi: 10.2307/2531734 3233245

[34] Hanley JA, Negassa A, Edwardes MD, Forrester JE. Statistical analysis of correlated data using generalized estimating equations: an orientation. Am J Epidemiol. 2003;157(4):364–75. doi: 10.1093/aje/kwf215 12578807

[35] Albert PS, McShane LM. A Generalized Estimating Equations Approach for Spatially Correlated Binary Data: Applications to the Analysis of Neuroimaging Data. Biometrics. 1995;51(2):627. doi: 10.2307/2532950 7662850

[36] Chiang CY, Slama K, Enarson DA. Associations between tobacco and tuberculosis. Int J Tuberc Lung Dis. 2007;11(3):258–62. 17352089

[37] McCarthy DE, Piasecki TM, Lawrence DL, Jorenby DE, Shiffman S, Fiore MC, et al. A randomized controlled clinical trial of bupropion SR and individual smoking cessation counseling. Nicotine Tob Res. 2008;10(4):717–29. doi: 10.1080/14622200801968343 18418793

[38] Zhou L, Guo K, Deng X, Shang X, E F, Xu M, et al. Effects of different combined behavioral and pharmacological interventions on smoking cessation: a network meta-analysis of 103 randomized controlled trials. J Public Health (Berl). 2023;33(9):1971–81. doi: 10.1007/s10389-023-02160-4

[39] Li Y, Lee Y, Port FK, Robinson BM. The impact of unmeasured within- and between-cluster confounding on the bias of effect estimatorsof a continuous exposure. Stat Methods Med Res. 2019;29(8):2119–39. doi: 10.1177/0962280219883323 31694489

